# Integrated Interventions for Executive Function Deficits in Children with Autism Spectrum Disorder: From Cognitive Training to Neuroregulation

**DOI:** 10.31083/AP40062

**Published:** 2026-02-26

**Authors:** Liuyan Zhu, Dan Yao

**Affiliations:** ^1^Department of Pediatric Health Care, Children’s Hospital, Zhejiang University School of Medicine, National Clinical Research Center for Children and Adolescents’ Health and Diseases, 310003 Hangzhou, Zhejiang, China

**Keywords:** Autism Spectrum Disorder, executive function, cognitive training, children

## Abstract

Autism Spectrum Disorder (ASD) is classified as a neurodevelopmental disorder primarily characterized by difficulties in social interaction, restricted interests, and repetitive behaviors. Advances in neuropsychological research have highlighted the crucial role of executive function (EF) deficits in children with ASD and their impact on the core symptoms of the disorder. EF encompasses higher-order cognitive processes, including working memory, cognitive flexibility, and inhibitory control. Given that EF deficits represent a significant cognitive impairment in this population, the variability in clinical intervention outcomes underscores the need for targeted strategies informed by underlying neural mechanisms. This narrative review explores the current research landscape on EF deficits in children with ASD. It synthesizes empirical findings related to cognitive and motor training, neuromodulation techniques, and collaborative interventions involving families and schools. The aim is to provide theoretical and practical guidance for enhancing EF and improving the quality of life of children with ASD.

## Main Points

1. Executive function deficits in autistic children are modifiable; targeted interventions during the preschool to early elementary years yield significant and sustained improvements.

2. Both physical-cognitive training and neuromodulation significantly improve executive function, with effects lasting weeks when dosage and targeting are optimized.

3. Sustaining these improvements necessitates strong family-school integration.

4. Future trials should adopt personalized, long-term, multi-site designs.

## 1. Introduction 

Autism Spectrum Disorder (ASD) is a multifaceted neurodevelopmental disorder characterized by impairments in social communication, repetitive behaviors, and restricted interests [1]. These symptoms typically emerge in early childhood and persist into adolescence and adulthood, often leading to long-term challenges in academic achievement, vocational adaptation, and independent living [2]. The precise cause of ASD is still unknown, but early environmental influences and genetic predispositions likely play a critical role in its development [3]. Epidemiologically, the prevalence of ASD has shown a gradual upward trend globally. According to the latest data from the Centers for Disease Control and Prevention, the prevalence of ASD among children aged 8 years in the United States has reached 1 in 36 with a male-to-female ratio of approximately 4:1 [4]. A systematic review and meta-analysis focusing on studies from 2017 to 2023 in mainland China reported a pooled prevalence of 7 cases per 1000. The analysis highlighted a significant male predominance, evidenced by a male-to-female ratio of 5:1 [5]. Notably, individuals with ASD also face an increased risk of comorbidities, including attention deficit hyperactivity disorder (ADHD), anxiety disorders, depression, and even early-onset dementia in adulthood, which further exacerbates the burden on families and society [6]. Given these clinical and public health challenges, exploring effective intervention strategies targeting ASD-related impairments has become a critical priority in neurodevelopmental research.

As research into ASD has advanced, there has been a growing focus on the role of executive function (EF) in children with ASD. EF refers to a range of high-level cognitive processes, including working memory, inhibitory control, and cognitive flexibility [7]. Developmentally, EF undergoes a protracted maturation process spanning childhood, adolescence, and early adulthood. Inhibitory control, the earliest maturing component of EF, begins to develop in infancy and reaches a critical milestone around 4–5 years of age, when children can consistently suppress prepotent responses [8]. Inhibition control is classified into two categories: response inhibition and interference inhibition. In general, children’s capacity for response inhibition evolves and improves as they grow older. In their study, Wang *et al*. [9] recruited 90 children aged 9 to 12 to participate in training with the spatial-Stroop serious game “Jungle Crossing”. Following the intervention, the participants in the intervention group showed a significant reduction in interference scores on both the spatial-Stroop and Flanker tasks. Importantly, these gains were maintained for one month after training. However, no significant improvements were observed in the Go/no-go task. This evidence suggests that interference suppression and response inhibition may follow separate developmental pathways [9]. Cognitive flexibility, by contrast, shows rapid growth between the ages of 6 and 9, reaching near full maturity between ages 10 and 12, and continues to develop throughout adolescence, peaking around the age of 21 [10]. Nonetheless, the academic community holds differing views on the development of cognitive flexibility in children. Doğru *et al*. [11] observed that traditional viewpoints posited that cognitive flexibility emerges around ages 3 to 4, typically measured using tasks such as the Dimensional Change Card Sort (DCCS). Conversely, recent theories contend that cognitive flexibility is not a discrete ability that abruptly appears during preschool years; instead, it is a gradual expression of controlled, goal-directed behavior that develops progressively from birth to age 6 [11].

In an intriguing study, a longitudinal analysis involving 12,330 English-speaking children aged 5 to 11.5 in the United States assessed working memory capacity (WMC) through a Digit Span Backward task. The findings revealed that WMC development exhibits a curvilinear pattern, showing a continuous increase with a progressively slowing rate of growth. Notably, the average rate of increase is most rapid every six months before reaching age 8, whereas post-age 8, the rate of growth significantly diminishes [12]. It is worth mentioning that EF continues to develop during childhood and adolescence, reaching full maturity in early to mid-adulthood, typically around the age of 20, and then gradually declines once adulthood begins, particularly after the age of 30 [13, 14]. Study has shown that while children and adolescents with ASD exhibit significant delays in in EF across core domains, compared to their typically developing peers, their age-related EF development trajectories are largely similar [15]. However, individuals with ASD frequently encounter challenges in cognitive flexibility, heightened social difficulties, and increased restrictive behaviors, as well as symptoms of anxiety and depression [16, 17, 18]. These findings provide valuable insights into the EF deficits observed in children with ASD, emphasizing the necessity for clinical interventions that promote the development of these cognitive functions.

The primary theoretical framework for EF is Baddeley’s working memory model [19], which includes four fundamental components, the phonological loop, the visuospatial sketchpad, the central executive system, and the episodic buffer. These components function collaboratively to store, process, and regulate information. Among them, the central executive system is paramount, as it governs controlled processing. Its responsibilities include activating long-term memory, coordinating the subsystems, regulating task switching and strategy selection, and managing attention and inhibitory control [20]. In clinical practice, one of the most widely used tools for assessing working memory is the digit span task. Inhibitory control is typically evaluated using tasks such as the Go/no-Go test [21] and Stroop tests [22], which are designed to measure how effectively individuals maintain focus and suppress irrelevant responses in the presence of distractions. Cognitive flexibility, another essential aspect of EF, is frequently assessed through tasks like the Wisconsin Card Sorting Test [23] and the DCCS [24]. These assessments provide valuable insights into the cognitive flexibility of individuals with ASD, enhancing our understanding of their performance in this domain.

In addition to experimental tasks, behavioral rating scales are a prevalent method for evaluating EF in children. One of the most frequently utilized scales is the Behavior Rating Inventory of Executive Function Preschool Version (BRIEF-P), which is specifically designed to assess the EF of preschool-aged children with ASD. A higher total score on the BRIEF-P indicates a greater degree of impairment in EF [25]. These experimental paradigms are crucial in psychological and neuroscience research, providing critical insights into the underlying mechanisms of EF and its role in everyday cognitive functioning. By employing these various measurement approaches, researchers can comprehensively assess the various components of EF in children with ASD.

## 2. Abnormalities in EF in Children With Autism and Their Impact on Core Symptoms

In children with ASD, EF deficits are not only highly prevalent but also closely intertwined with the core symptoms of the disorder [26]. Studies (Each group comprises 22 individuals aged 11 to 20 years, with a total duration of approximately 10 to 14 days) utilizing eye-tracking technology have shown that children with ASD generally have lower accuracy in reading tasks. Unlike the control group, which modifies its reading strategies according to varying objectives, children with ASD display minimal changes in their reading behaviors [27]. Additionally, the research highlights that planning ability, an essential aspect of EF, can predict differences in reading time, implying that children with ASD encounter significant challenges in attaining a thorough understanding of reading tasks. This difficulty is strongly associated with their EF impairments. Therefore, it is clear that these reading difficulties may indicate broader cognitive control challenges experienced by children with ASD. A study (The study enrolled 100 children aged 6–12 years, divided into an ASD group (n = 62, age 6.8–12.8 years) and a control group. Within the ASD group, 27 participants had comorbid ADHD, and 22 were taking psychotropic medications (including stimulants and antipsychotics). The design was cross-sectional, with a single data-collection session lasting 90–120 minutes) using the Go/no-Go task found that cognitive control in children with ASD significantly decreased when they were presented with objects of interest [28]. This suggests that cognitive flexibility in children with ASD may be inversely related to their level of interest in certain objects. However, a comparative study (a total of 131 adolescents aged 10 to 16 were included in the study, divided into four groups. The ASD group had 41 participants, aged 11.33 to 15.67 years, with intelligence quotients (IQs) ranging from 54 to 129. The research used a cross-sectional design, and all data were gathered during a single test session lasting about 60 to 90 minutes) conducted by Carter Leno *et al*. [29], using Go/no-Go and task-switching tasks with typically developing children, those with ADHD, children with oppositional defiant disorder, and children with ASD yielded surprising findings. It found no significant differences in cognitive flexibility between the ASD group and the other groups [29]. Taken together, these findings suggest that while children with ASD may face challenges in inhibitory control and working memory, the complexities of their cognitive difficulties necessitate further exploration.

EF deficits can make it particularly challenging for children with ASD to navigate and adapt to social situations. Difficulties with inhibitory control and cognitive flexibility are often correlated with the repetitive behaviors and restricted interests observed in these children. Mechanistically, impaired inhibitory control contributes to the persistence of repetitive and stereotyped behaviors, as children with ASD may find it difficult to inhibit automatic, rigid responses. Cognitive flexibility, a vital cognitive skill, allows individuals to adjust their thoughts and actions based on situational context. This ability is crucial for effectively reacting to changes in the environment and for rapidly adapting to new rules, both of which are essential for managing the unpredictability of life [30]. However, research indicates that children with ASD face considerable obstacles in social interaction, reward processing, and emotional regulation. Dysregulation of dopamine in the midbrain-limbic pathway, along with imbalances in neurotransmitters such as γ-aminobutyric acid and serotonin, may contribute to difficulties in emotion management, anxiety, and related cognitive-behavioral abnormalities [31].

All in all, the evidence reviewed above indicates that EF deficits not only impede the social abilities of children with ASD, but may also exacerbate their challenges with emotional regulation and behavioral issues, thereby underscoring the intricate interplay between the core symptoms of autism and EF. Encouragingly, a recent study involving 117 children with ASD (the average age was 10.25 ± 1.481 years, and the participants were in grades 1 to 6. This study was observational, collecting data on indicators in a natural state through questionnaires and physical fitness tests) and 311 typically developing peers found that children with ASD exhibited significantly lower levels of both EF and social skills compared to their typically developing counterparts. The research further revealed a robust correlation between EF and social skills. There is optimism that enhancing physical fitness levels may concurrently improve both EF and social skill deficits among children with ASD [32]. Additionally, a study utilizing the Early Childhood Longitudinal Study-Kindergarten 2011 (ECLS-K:2011) dataset examined the development trajectories of working memory in approximately 310 children with autism (participants aged approximately 5 to 6 years were included and followed for 6 years, resulting in an average age of about 11 to 12 years at the end of the study. This was an observational longitudinal study that explored the trajectories of working memory development using natural tracking data from ECLS-K:2011, without any specific intervention designs) and 3410 typically developing children throughout their elementary education (spanning from kindergarten to fifth grade). The findings revealed substantial growth for both groups during early elementary school. However, children with autism experienced a prolonged phase of high plasticity that lasted until the end of second grade. Importantly, those who started with lower working memory levels exhibited rapid advancement from third to fifth grade, often classified as “late bloomers”, while the development pace of typically developing children began to slow down following the end of first grade. This underscores the critical need for tailored interventions for children with autism, with a particular emphasis on addressing the distinctive trajectory of “late bloomers” during grades three to five [33].

This review investigates current empirical findings regarding EF deficits in children with ASD. We will then critically evaluate the impacts of cognitive training, physical exercise, neuroregulation, and family-school collaborative interventions, with the goal of delivering evidence-based recommendations for future research and clinical applications.

## 3. New Strategies for Intervening in EF Deficits in Children With ASD

Various cognitive and behavioral interventions have recently been found to significantly enhance EF in children and adolescents with ASD. While EF naturally improves as individuals mature, evidence suggests that it can be further strengthened through specialized training [34, 35]. Therefore, active participation in these interventions can lead to meaningful progress in the development of EF in this population.

It’s noteworthy that both childhood and adolescence exhibit high levels of neural plasticity, rendering interventions during these developmental stages especially effective [36]. Throughout childhood, which includes both infancy and early childhood, the brain enters a distinctive phase characterized by “synaptic exuberance and diffuse network formation”. During this time, the excitability and plasticity of cortico-subcortical circuits associated with language, motor development, and essential social skills are significantly enhanced, with the establishment of synaptic connections strongly dependent on environmental stimuli. Offering abundant sensory experiences and basic skills training at this stage can effectively consolidate neural pathways, thereby laying a solid foundation for critical abilities in language, movement, and social interaction. As individuals transition into adolescence, the plasticity of the brain evolves from “diffuse proliferation” to “precise pruning”, primarily concentrating on the fronto-limbic circuitry that encompasses the amygdala and striatum. As a result, intervention strategies must adapt accordingly: advancing from the “enriched environment” model utilized during childhood to “functional optimization” in adolescence, moving from multisensory and skill-oriented stimulation toward focused training in emotional regulation and advanced cognitive processes [37].

The journey of EF development commences at birth, exhibiting marked growth particularly between the ages of 3 and 6, thereby designating the preschool years as a vital period for its development [38, 39]. Throughout this phase, children with ASD show gradual improvements in EF as they move from preschool to school age and this developmental pathway remains highly malleable [40]. By implementing well-designed intervention strategies and timing them appropriately, we can significantly enhance the EF of children and adolescents with ASD, thus fostering their overall development.

### 3.1 Intervention Methods Based on Exercise and Cognitive Training

The multi-pathway theoretical model for physical interventions aimed at facilitating EF development in children suggests that physical exercise serves as an effective mechanism for enhancing EF. A recent systematic review and meta-analysis demonstrate that exercise therapy can substantially and consistently enhance EF in children and adolescents with ASD. However, preschool-aged children (ages 3–7) did not exhibit significant improvements in EF following these interventions. The gains in working memory were minimal, and variables such as the duration of the exercise program, the type of exercise (including virtual reality training), and medication administration did not have a significant impact on the efficacy of the intervention [41]. Numerous studies have demonstrated that physical activity can effectively address EF deficits in children with ASD. For instance, Tse *et al*. [42] implemented a basketball skills training program involving 19 autistic children aged 8 to 12 years. After 12 weeks, the intervention group exhibited significant improvements in inhibitory control, while the control group showed no meaningful changes. Nonetheless, the intervention did not lead to any significant improvements in working memory for either group [42]. In another study, Ji *et al*. [43] investigated 100 children with ASD, randomly assigning them to a virtual training group, a physical exercise group, or a control group. Following six weeks of training, both the virtual training and physical exercise groups demonstrated improvements in EF. However, three weeks after the intervention concluded, EF levels in both groups declined, indicating that football-based interventions may have limitations regarding their long-term effects [43]. In a separate exercise intervention study [44], 34 children with ASD, aged 8 to 11 years, were randomly assigned to either a mixed martial arts (MMA) intervention group or a control group. The MMA group participated in 26 training sessions over a period of 13 weeks. The findings revealed that the MMA intervention led to significant improvements in EF, particularly in inhibitory control, working memory, and cognitive flexibility. However, the improvement in inhibitory control was less pronounced compared to the other dimensions of EF. In summary, while physical exercise has shown potential in enhancing EF among children and adolescents with ASD—particularly in aspects such as inhibitory control—the sustainability of these effects remains unclear. Further research is essential to identify the optimal parameters for such interventions to maximize their benefits over time.

Recent years have seen a growing interest in the impact of exercise interventions on EF in children with ASD. A notable study conducted by Ludyga *et al*. [45] examined a sample of 376 children and adolescents aged 5 to 18 years, comprising 174 individuals in the ASD group and 202 in a typical development control group. Utilizing Structural Equation Modeling (SEM), the researchers investigated the relationships between ASD, muscle strength-measured through Fitnessgram metrics including push-ups, sit-ups, and trunk lifts—Body Mass Index, EF, and information processing. After adjusting for confounding variables such as age and gender, the findings revealed that ASD was significantly associated with decreased muscle strength and impaired EF. Importantly, muscle strength exhibited an independent positive predictive relationship with both information processing and EF. This beneficial association was identified solely within the ASD group, suggesting that greater muscle strength correlates with enhanced EF and information processing capabilities in children with ASD. These findings underscore the potential benefits of interventions aimed at increasing muscle strength to mitigate EF deficits and improve information processing in this population [45].

Furthermore, Greco and De Ronzi [46] conducted a study involving 28 children aged 8 to 11 years diagnosed with mild to moderate ASD. Utilizing a randomized controlled design, the participants were matched and divided into either an intervention group or a control group. The intervention group engaged in a 12-week karate training program, participating in classes twice weekly for 45 minutes each session. The effectiveness of the intervention was evaluated using the Social Skills Improvement System Rating Scale and the BRIEF-P, with assessments completed by the parents. The findings revealed that the twelve-week karate program resulted in significant enhancements in social skills, particularly in communication and cooperation among children with ASD. Additionally, the program effectively diminished problem behaviors such as aggression and anxiety, while promoting improvements in EFs, including inhibitory control and working memory. Crucially, all observed enhancements displayed a large effect size [46]. The latest review underscores that, for the ASD population—predominantly composed of children aged 3 to 12 years, there are no absolute contraindications against any form of exercise. Various individual sports, such as martial arts and swimming, as well as team sports including basketball and soccer, have been shown to significantly improve social skills, motor abilities, emotional regulation, and EFs (including working memory and cognitive flexibility) in individuals with ASD. Notably, martial arts (karate and judo) and equestrian therapy demonstrate particularly strong therapeutic effects. It is recommended that interventions follow individualized principles, suggesting participation 2 to 3 times a week, with each session lasting between 45 to 60 minutes, maintained over a minimum of 12 weeks [47]. Additionally, Tao *et al*. [48] examined the effects of four different physical activity interventions-Aerobic Exercise (AE), Mind-body Exercise, Exergaming, and Multi-component Physical Activity (MPA)-compared to Usual Care on cognitive function (encompassing attention, memory, and EF) and acceptability in children and adolescents with neurodevelopmental disorders aged 5 to 17 years. Their results indicated that Exergaming resulted in significant improvements in memory and EF overall. However, it was ineffective for enhancing EF in individuals with ASD and did not benefit attention or memory in those with ADHD. In contrast, MPA consistently improved cognitive function across both ADHD and ASD populations, emerging as the only intervention demonstrating efficacy across different subtypes. Conversely, AE did not produce significant benefits in any cognitive domain [48].

Beyond physical exercise, cognitive training has demonstrated promising effects as well. Macoun *et al*. [49], developed a game-based cognitive training program called “Caribbean Quest”, grounded in metacognitive teaching theory. They implemented an eight-week intervention involving 20 children with ASD, aged 6 to 12 years, featuring individualized computerized training sessions that lasted 30 minutes each week. The findings indicated substantial enhancements in the participants’ working memory and cognitive fluency [49]. These studies collectively highlight the positive influence of both physical exercise and cognitive training on EF in children with ASD, offering critical insights for the development and refinement of future intervention programs.

In summary, we present the following findings: Table 1a (Ref. [42, 43, 44, 45, 46, 49]) provides a comprehensive overview of the various intervention types discussed earlier, while Table 1b investigates potential factors contributing to the inconsistent improvements in EF linked to different intervention strategies. Furthermore, informed by the current body of research, we delineate possible avenues for future investigations.

**Table 1a.** **All human studies cited in this section received prior approval from the relevant institutional review board or equivalent ethics committee, along with written informed consent from a parent or legal guardian**.

Type of training	Number of individuals with ASD, age	Intervention duration	Subcomponents of EF improved	Ref.
A basketball skills training program	19, 8–12 years old	12 weeks, 24 sessions (2 sessions/week, 45 minutes/session), total duration: 18 hours	Improvement in inhibitory control rather than working memory	[42]
A virtual training group, a physical exercise group (Football)	100, 12–15 years old	6 weeks, 3 times per week, 1 hour each session. Follow-up conducted 3 weeks after training completion	Improvements in EF; however, three weeks after the intervention ended, both groups showed a decline in EF levels.	[43]
Mixed martial arts	34, 8–11 years old	2 sessions/week, 45 minutes/session, 13 weeks, total duration: 26 sessions	Improvements in EF, especially in inhibitory control, working memory, and cognitive flexibility. However, the degree of improvement in inhibitory control was not as significant as that of the latter two.	[44]
Utilizing SEM to explore the relationships among ASD, muscle strength, EF, and information processing	174, 5–18 years old	Completed questionnaires SDQ, BSMSS, and PPS, along with diagnostic interviews, NIH Toolbox cognitive tasks, and FitnessGram physical fitness tests	ASD is significantly associated with a decline in muscle strength and impaired EF; higher levels of muscle strength in children with ASD are correlated with better EF and information processing abilities.	[45]
Karate Training	28, 8–11 years old	12 weeks, 2 times per week, 45 minutes each session	Enhancing EFs, including inhibitory control and working memory.	[46]
A game-based cognitive training program called “Caribbean Quest”	20, 6–12 years old	Featuring one-on-one computerized training sessions that lasted 30 minutes each week, for a total of 8 weeks.	Improvements in the participants’ working memory and cognitive fluency	[49]

ASD, Autism Spectrum Disorder; EF, executive function; SEM, Structural Equation Modeling; SDQ, Strengths and Difficulties Questionnaire; BSMSS, Barratt Simplified Measure of Social Status; PPS, Peterson Puberty Scale; NIH, National Institutes of Health.

**Table 1b.** **The potential reasons for the inconsistent improvements in EF**.

Dimension	Specific factors	Possible impact on efficacy	Recommendations
Intervention duration and program variations	Duration: Ranges from 6 to 13 weeks. Frequency: 2 to 3 times per week. Each session lasts 30 to 60 minutes.	The time-effect curve is unclear, short durations may make it difficult to induce lasting neural plasticity.	Future studies should systematically investigate a stepwise escalation design with a duration of at least 24 weeks and a total time of at least 36 hours.
Content variation	Basketball/Football (Open Skills) vs Martial Arts/Karate (Cognitive-Motor Coupling) vs Virtual Training (Task-Specific).	The varying demands on working memory, inhibitory control, and cognitive flexibility result in inconsistent improvements across subcomponents.	Establish a ‘Cognitive Load Classification’ framework to match targeted EF components.
Differences in participant traits (age, intelligence quotient (IQ), ASD severity)	Age: The range spans from 5 to 18 years, which is too broad. Functioning Level: High-functioning ASD vs Low-functioning ASD. Children with ASD have varying IQ levels.	High-functioning/High IQ children with ASD are more likely to understand instructions and benefit more from interventions. Low-functioning children may have limited gains due to difficulties in comprehension.	Stratified randomization based on age, IQ, and symptom severity, with reporting of effect sizes in relation to baseline characteristics.
Research design differences	Most studies are cross-sectional or have short follow-ups (3–6 weeks), lacking long-term longitudinal tracking.	It is difficult to determine whether the effects are sustained or how long they may last.	Design a 6–12 month longitudinal follow-up, incorporating Ecological Momentary Assessment to monitor daily EF performance.
Influence of family dynamics and socioeconomic status	Parental involvement, family socioeconomic status (SES) and parents’ educational level.	High SES and parental involvement may enhance adherence and amplify effects.	Collect indicators of SES and parental involvement for moderation/mediation analysis.

For example, the basketball trial was approved by the Education University of Hong Kong Ethics Committee [42]. The football study received approval from Northeastern University, China (EC2021B001) [43]. The MMA intervention adhered to the 1964 Helsinki Declaration [44], while the karate program complied with ethical standards set by the responsible institutional committee on human experimentation and the Helsinki Declaration. Furthermore, the “Caribbean Quest” program was conducted with approval from the University of Victoria Human Ethics Board and in accordance with Tri-Council Human Ethics guidelines [49].

First, there was considerable variability in training doses among studies, with total intervention durations ranging from 18 hours (for basketball, football, and karate) to under 5 hours (for virtual cognitive training), and none exceeding 20 hours. In the absence of a clear defined dose-response relationship, these relatively short durations may explain the modest or non-significant improvements in working memory, as well as the rapid decline observed three weeks following football training. The effectiveness of EF interventions in ASD appears to be closely related to the timing of the intervention, including factors such as total duration, frequency, and overall training dose.

Second, the cognitive demands presented by different activities, ranging from open-skill ball games to martial arts sequences and computerized tasks, engage various sub-domains of EF. For example, basketball and football predominantly focus on open skills, which require high levels of inhibitory control while placing comparatively lower demands on working memory updating. Conversely, martial arts practices, such as karate, concentrate on action sequence memory and rule-switching, simultaneously stimulating both inhibitory control and working memory, resulting in meaningful improvements in these areas. Although virtual cognitive training (e.g., Caribbean Quest) involved a total dosage of only 4 hours, it was specifically tailored to target “working memory and cognitive fluency”, yielding pronounced effects on those specific cognitive components.

Third, the characteristics of participants varied widely, with ages ranging from 5 to 18 years. Only a limited number of trials stratified outcomes according to IQ or ASD severity, indicating that higher-functioning individuals likely benefited more from the interventions due to better comprehension of the instructions provided.

Fourth, most trials reviewed here employed cross-sectional designs or featured brief follow-up periods (less than 6 weeks), which limits the ability to draw conclusions regarding the long-term sustainability of effects. Additionally, factors such as parental involvement and family socioeconomic status—often underreported—may influence adherence to interventions and affect the quality of training received. The lack of a phase involving “family-school joint reinforcement” in existing studies could further contribute to a rapid regression in functional gains shortly after training concludes.

To enhance future research, several methodological improvements should be implemented, including standardized dose-escalation protocols, stratified sampling based on age, IQ, and severity of ASD, as well as longitudinal follow-ups of six months using ecological momentary assessment. Furthermore, systematic evaluation of family-level moderators would facilitate a clearer understanding of causal mechanisms and support the optimization of individualized interventions.

Despite progress in utilizing exercise and cognitive training to enhance EF in children with ASD, significant challenges continue to exist, including variability in long-term outcomes and modest improvements in targeted cognitive areas. The latest advancements in neuroscience have introduced neuroregulation technologies, which present new opportunities for refining interventions aimed at this population. In the following sections, we will discuss how the integration of neuroregulation technologies can enhance intervention strategies, ultimately providing more effective support for the development of EF in children with ASD.

### 3.2 Intervention Methods Combined With Neuroregulation Technologies

Recently, neuroregulation technologies have emerged as a promising strategy for improving EF in individuals with ASD, offering novel therapeutic alternatives for those who respond poorly to traditional behavioral interventions. These technologies work by directly modulating aberrant neural activity and specifically targeting the mechanisms associated with neural dysfunction. Among these techniques, repetitive transcranial magnetic stimulation (rTMS)—a non-invasive method—has attracted considerable interest in the realm of ASD intervention research. By manipulating cortical excitability through electromagnetic induction, rTMS has established itself as a key focus of inquiry. The mechanisms underpinning rTMS include long-term potentiation and long-term depression. High-frequency stimulation (5–20 Hz) is aimed at the dorsolateral prefrontal cortex (DLPFC), which has been linked to significant enhancements in working memory and cognitive flexibility. In contrast, low-frequency stimulation (≤1 Hz) serves to inhibit overactive areas such as the primary motor cortex, helping to mitigate repetitive and stereotypical behaviors [50]. Research indicates that the DLPFC is crucial for effective ASD interventions because it’s responsible for processing language, auditory input, and spatial information in working memory. Notably, individuals with ASD display markedly lower levels of DLPFC activation when performing tasks compared to their neurotypical counterparts [51]. Encouragingly, different types of transcranial magnetic stimulation have proven effective in improving clinical symptoms while also demonstrating both the feasibility and safety of enhancing EF in adult ASD patients [52], thereby guiding future research directions in this promising field.

A notable study in the field of low-frequency neurofeedback involved 35 children with ASD, aged 7 to 17 years. During a 10-week intervention period, participants attended neurofeedback training sessions three times a week, culminating in a total of 30 sessions. Objective measures were obtained through various assessment tools, including the Flankers inhibitory control and attention test, as well as the DCCS task. The results revealed marked improvements in inhibitory control, attention, cognitive flexibility, processing speed, and working memory, with these gains maintained during a follow-up assessment conducted two months later [53]. Numerous randomized controlled trials have further corroborated the beneficial impact of repetitive rTMS on EF in children with ASD. In a study led by Ameis *et al*. [54], 20 sessions of high-frequency (20 Hz) stimulation were administered to the left DLPFC for 30 minutes each to children aged 7 to 17 years. Results demonstrated significant improvements in working memory and inhibitory control, with effects persisting for four weeks post-treatment [54]. Another study by Casanova *et al*. [55] applied a low-frequency stimulation protocol, and following 10 sessions of left DLPFC stimulation, considerable improvements were noted in event-related potential measures of attention in children aged 8 to 17 years, with benefits sustained for six weeks. Additionally, rTMS targeting brain areas such as the dorsomedial prefrontal cortex and posterior superior temporal gyrus has shown promise in improving social skills and diminishing repetitive behaviors, although substantial individual variability exists. Stimulation of the dorsolateral prefrontal cortex is also associated with enhancements in EF and emotional symptoms, albeit requiring further large-scale validation [56, 57]. In terms of safety, the occurrence of adverse events from rTMS in children and adolescents is reported to be between 3.4% and 10.11%, with transient headaches and neck pain being the most frequently observed side effects, severe adverse events are infrequently documented [58].

Due to its cost-effectiveness and ease of use, transcranial direct current stimulation (tDCS) has gained considerable attention as an alternative to rTMS. This non-invasive technique modifies cortical excitability by employing anodal stimulation to enhance neural activity or cathodal stimulation to inhibit it, thereby affecting neural plasticity. Study has found that following anodal tDCS intervention, children with ASD exhibited significant decreases in their scores for social communication and motivation on the Social Responsiveness Scale, as well as marked improvements in various dimensions of the Autism Behavior Checklist, including sensory behaviors and social interactions [59]. In contrast, the outcomes from cathodal tDCS interventions did not reach statistical significance, likely due to the small sample size involved. Another study applied 20 minutes of anodal tDCS stimulation to both children with ASD and a sham stimulation control group followed by an analysis of resting-state electroencephalogram (EEG) data. The results revealed that tDCS significantly enhanced brain network flexibility and increased the number of modules in children with ASD, while modulating functional connectivity across frequency bands including alpha, beta, and gamma. The findings also highlighted that tDCS had a favorable safety and tolerability profile [60]. A wealth of studies suggest that tDCS is generally safe and well-tolerated both in research settings and for at-home use, with mild side effects such as tingling and itching being commonly observed [61, 62]. Large-scale datasets indicate that serious adverse effects are rare.

Nonetheless, the use of tDCS in children with ASD raises ongoing discussions. Although it is usually considered safe for pediatric populations, the long-term implications of tDCS on brain development require further scrutiny [63, 64]. Some research has suggested that the effectiveness of tDCS may be moderated by individual genetic factors, including the catechol-*O*-methyltransferase (*COMT*) gene (rs4680) Val158Met polymorphism [65]. Particularly, multi-session tDCS, where a negative electrode is positioned over the left DLPFC and the right supraorbital area in conjunction with cognitive remediation training, has been correlated with enhancements in social function among individuals with ASD, potentially linked to increased functional connectivity in the right medial prefrontal cortex, which is essential for flexible processing of social information [66]. Encouragingly, recent research [67] indicates that both rTMS and tDCS can effectively and safely enhance EF and reduce repetitive stereotypical behaviors in children and adolescents with ASD who possess an IQ of 65 or above. The findings suggest that tDCS is particularly effective in improving social communication, while rTMS excels at mitigating repetitive and atypical behaviors. Both modalities primarily target the DLPFC and operate by modulating its excitability, thus aiming to restore the integrity of fronto-temporal social network and the fronto-striatal behavior regulation network, ultimately fostering greater neural plasticity.

Despite these advancements, notable differences exist in the parameters of the interventions, and most follow-ups are restricted to 14 weeks or less, leaving the long-term effects largely unexamined. Fortunately, innovative closed-loop regulation technologies, including real-time functional magnetic resonance imaging neurofeedback and EEG neurofeedback, are emerging as promising avenues. One investigation [68] involving 13 ASD patients without intellectual disabilities and 17 healthy controls found that speech working memory neurofeedback training, implemented via the left DLPFC, allowed ASD patients to regulate DLPFC activity. This training subsequently enhanced connectivity between the DLPFC and the motor cortex, compensating for pre-existing deficits in the premotor area’s activity, thereby bringing the difference in brain activity levels between the two groups and offering a novel strategy for EF rehabilitation. While neuroregulation technologies represent a new biomedical platform for targeting EF interventions in ASD, they remain in the clinical validation phase and require extensive randomized controlled trials to solidify their evidence base. Moreover, although these techniques exhibit considerable potential in both laboratory and clinical settings, it’s crucial that ASD interventions are not exclusively reliant on any single technological approach. In fact, the rehabilitation efforts for individuals with ASD must be approached as a systemic undertaking, requiring coordinated strategies that engage both home and educational environments.

The subsequent discussion will focus on how to formulate a comprehensive intervention model within family and school settings, integrating neuroregulation technologies alongside environmental support to develop a holistic and tailored rehabilitation framework for individuals with ASD.

### 3.3 Comprehensive Intervention Model in Family and School Environments

Existing intervention frameworks advocate for the integration of EF improvement strategies for children with ASD into their daily lives, extending beyond traditional clinical or laboratory environments to include both home and school settings. This ecological approach not only increases the accessibility and sustainability of such interventions but also enhances their overall effectiveness by fostering cooperation across different environments. In a recent study, researchers explored the impact of three forms of physical activities on EF in children with autism: Xbox Kinect-based active video games (AVG), sedentary video games (SVG), and brisk walking. The study involved children with autism aged 8 to 11, who were randomly assigned to one of the three groups, each engaging in a 20-minute session of their designated activity. Results indicated that the AVG group exhibited a significant improvement in EF test scores post-intervention, outperforming the SVG group substantially. This outcome serves as robust empirical evidence for the effectiveness of technology-assisted interventions implemented in home environments [69].

In child development research [70], the positive impact of supportive parenting on children’s long-term adjustment has been robustly supported by longitudinal studies. A significant longitudinal study in Norway followed 455 families and found that fathers who exhibited higher levels of supportive parenting—defined by sensitivity, warmth, encouragement, and involvement—when their children were 36 months old, led to fewer instances of hyperactivity and impulsivity when those children reached first grade. The complementary roles of mothers and fathers are crucial, when one parent’s support is insufficient, strong support from the other parent can significantly alleviate externalizing behaviors and enhance social skills, highlighting the importance of cooperative parental intervention.

For children with autism, EF often lags behind their neurotypical peers. The rationale for integrated family-school interventions is fundamentally rooted in family systems theory, providing valuable insights for the design of EF training programs tailored to these children [70]. The family unit serves as a primary setting for a child’s development, offering distinct advantages for intervention. Firstly, parents can engage their children in structured play, household responsibilities, and various daily routines, thereby promoting the enhancement of the child’s EF. Secondly, the home environment enables personalized interventions that align with the specific needs of the child. Furthermore, interventions involving both parents and children not only improve the child’s EF but also enrich the quality of their interactions. Wood *et al*.’s research [71] demonstrated that for children with ASD, adapting cognitive-behavioral therapy to meet their unique needs, such as integrating parental involvement, providing social communication support, and incorporating context-specific strategies into structured treatment, yields more favorable outcomes in anxiety reduction and adaptive functioning. Autistic children often face difficulties in emotional regulation and social adaptation. Thus, when parents implement supportive strategies, they not only enhance the child’s EF but also augment and enrich interventions provided in school, effectively addressing the shortcomings of any single context. Therefore, any EF intervention framework constructed around the comprehensive intervention model should emphasize the systematic empowerment of parents as co-interveners, ensuring a holistic and finely-targeted educational approach.

As a fundamental environment in a child’s life, school plays a significant role in the comprehensive intervention model. Educators can seamlessly incorporate EF training into everyday classroom activities by employing strategies such as defining classroom rules and segmenting tasks into smaller, manageable components. Additionally, fostering robust collaboration between home and school aids in sustaining consistency in intervention strategies, which in turn amplifies their effectiveness and facilitates generalization across different contexts. This integrated, multi-environmental approach offers more comprehensive and lasting support for the development of EF in children with ASD.

The cross-environmental collaborative intervention model represents the forefront of interventions designed for children with ASD. By establishing a collaborative network among families, and therapists, social work professionals, and public health teams, this model promotes the sharing of data and the coordination of strategies, aligning individualised care, population-level support, and evidence-based decision-making, ensuring both consistency and sustainability in interventions. Leveraging accessible digital platforms to track a child’s EF performance and independent skill execution in real-time across home and community environments enables ongoing, data-informed adjustments to intervention plans. Supported by empirical research, this integrated approach, bolstered by virtual coaching for caregivers to maintain intervention fidelity. These studies indicate that this integrated approach has a notably positive effect on improving cognitive flexibility and emotional regulation and independent play behaviors in children with ASD [72, 73].

Furthermore, it is imperative to emphasize interdisciplinary research collaborations that encourage deep integration across the fields of education, medicine, and psychology. Particular emphasis should be placed on investigating the role of advanced technologies, including big data analysis and artificial intelligence, in evaluating and addressing the executive functions of children with ASD. Ensuring access to scientific expertise and technical solutions is vital for supporting enhancements in their EF skills. This organized, multi-faceted intervention framework not only meets the developmental needs of children with ASD but also mirrors the growing trend towards holistic rehabilitation services, thereby presenting promising avenues for improving the social adaptability and overall quality of life for these children.

## 4. Conclusions

This review synthesizes emerging evidence on EF deficits in children with ASD, emphasizing a paradigm shift from isolated interventions to integrated, multi-modal strategies that combine cognitive training, neuromodulation, and family-school ecosystems. The findings indicate that the preschool-to-early-elementary years represent a critical period of high plasticity, during which targeted EF interventions can result in the most significant and enduring improvements.

Future research should focus on four primary trajectories. Firstly, it is critical to establish connections between multidimensional biomarkers, such as EEG signatures and genetic polymorphisms like *COMT* gene Val158Met, and treatment outcomes. This approach would facilitate the precise matching of interventions, including rTMS, tDCS, or cognitive-motor training protocols with individual profiles. Secondly, studies should be conducted over a duration of at least 24 weeks, incorporating a total training dosage of at least 36 hours, to systematically compare the efficacy of multi-component physical programs against neuro-modulation-augmented training methods. Moreover, there is a pressing need to create digital platforms that integrate real-time monitoring of EF, tools for home-school coordination, and adaptive guidance to maintain intervention benefits beyond clinical environments. At the same time, it is important to incorporate parent-co-training modules into existing special education frameworks. Trials should also expand into under-researched areas, such as remote mountainous villages in China, and include adolescent and adult samples, while ensuring that the content of interventions is adapted to local parenting styles and educational practices.

**Fig. 1.**
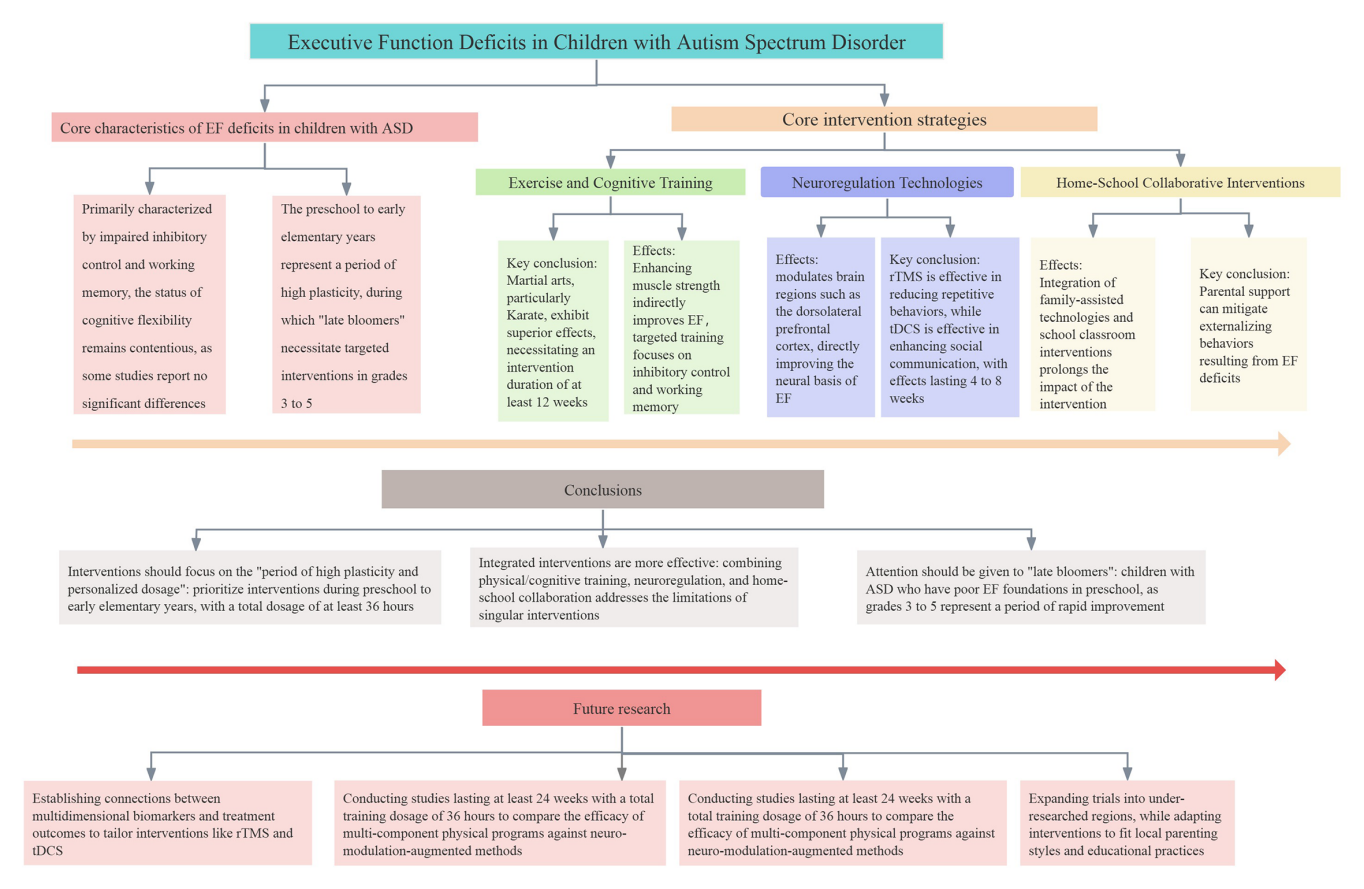
**Core framework, key findings, and future directions of integrated interventions for EF deficits in children with ASD**.

By integrating cognitive-motor interventions with neuroregulation technologies and a collaborative family-school approach—amplified via internet-based platforms, we can systematically enhance the executive functions of autistic children, mitigate their core symptoms, and thereby unlock the lifelong developmental potential inherent in every individual on the spectrum. Fig. 1 provides a schematic overview of these integrated pathways and outlines future research priorities.
